# Using non-invasive brain stimulation to augment motor training-induced plasticity

**DOI:** 10.1186/1743-0003-6-8

**Published:** 2009-03-17

**Authors:** Nadia Bolognini, Alvaro Pascual-Leone, Felipe Fregni

**Affiliations:** 1 Berenson-Allen Center for Noninvasive Brain Stimulation, Beth Israel Deaconess Medical Center, Harvard Medical School, Boston, USA; 2 Department of Psychology, University of Milano Bicocca, Milano, Italy; 3 Institut Guttmann de Neurorehabilitacio, Universitat Autonoma de Barcelona, Barcelona, Spain

## Abstract

Therapies for motor recovery after stroke or traumatic brain injury are still not satisfactory. To date the best approach seems to be the intensive physical therapy. However the results are limited and functional gains are often minimal. The goal of motor training is to minimize functional disability and optimize functional motor recovery. This is thought to be achieved by modulation of plastic changes in the brain. Therefore, adjunct interventions that can augment the response of the motor system to the behavioural training might be useful to enhance the therapy-induced recovery in neurological populations. In this context, noninvasive brain stimulation appears to be an interesting option as an add-on intervention to standard physical therapies. Two non-invasive methods of inducing electrical currents into the brain have proved to be promising for inducing long-lasting plastic changes in motor systems: transcranial magnetic stimulation (TMS) and transcranial direct current stimulation (tDCS). These techniques represent powerful methods for priming cortical excitability for a subsequent motor task, demand, or stimulation. Thus, their mutual use can optimize the plastic changes induced by motor practice, leading to more remarkable and outlasting clinical gains in rehabilitation. In this review we discuss how these techniques can enhance the effects of a behavioural intervention and the clinical evidence to date.

## Introduction

Motor impairments following stroke or traumatic brain injury (TBI) are the leading cause of disability in adults. More than 69% of all stroke survivors experience lasting functional motor impairments in the upper limbs and approximately 56% continue to complain of marked hemiparesis as long as 5 years post-stroke [1-5]. Such losses in function can severely impact quality of life and the functional independence in numerous activities of daily living [4,5]. Similarly, after TBI, fine and gross motor deficits are frequently observed. Complementary impairments such as ataxia, movement disorders and vestibular impairments, can also potentially affect motor functioning in TBI. Moreover, other factors such as multiple trauma, resulting in musculo-skeletal and peripheral nervous system injury, also complicate the recovery of motor functions in these patients [6].

Although some degree of recovery may occur spontaneously, there is strong evidence that intensive practice is essential in order to substantially promote motor recovery [7-9]. As shown by several neurobehavioral discoveries in animals and humans, such experience-dependent change can occur at multiple levels of the central nervous system, from the molecular, to the synaptic level of cortical maps and large-scale neural networks [10,11].

Standard motor therapies involve different approaches aimed at improving motor functions by minimising impairment or developing suitable adaptation strategies. For instance, neurofacilitation techniques are aimed at retraining motor control by promoting normal (recruitment of paretic muscles) while discouraging abnormal movement or muscle tone. Different facilitation approaches have been developed, including cutaneous/proprioceptive, weight bearing, proximal pre-innervation, and contralateral pre-innervation [12]. Task-specific training is aimed at improving skill in performing selected movement or functional tasks: examples of this type of treatment are index finger tracking [13] or the combination of task-specific motor training with the inhibition of ipsilesional sensorimotor cortex representation of the paretic upper arm by local anaesthesia [14]. Finally, task-oriented training aimed at retraining functional tasks by taking into account the interplay of different systems is another possible approach. For example, constrained-induced movement therapy (CIMT) combines intensive physical practice using the affected upper limb with restricted use of the unaffected upper limb in order to prevent its habitual compensatory utilization [15]. Bilateral arm training is instead based on the phenomenon of interlimb coupling, in which the movement patterns of the arms are similar when moving simultaneously [16,17]. Ongoing studies indicate that even mere action observation, activating the same cortical motor areas that are involved in the performance of the observed actions (i.e. action observation/execution matching system) can lead to a reorganization of the motor system resulting in an improvement of motor functions [18,19]. Other treatments have focused on the use of robotics [20,21], EMG-triggered stimulation [22], and motor imagery [23] (see for a review [24]).

Although there is little doubt that behavioural motor therapy clearly plays a role in promoting contra- and ipsi-lesional plastic changes after stroke, the functional outcomes are often of limited practical significance and after completing standard rehabilitation approximately 50–60% of patients still exhibit some degree of motor impairment and require at least partial assistance in activities of day living [24,25]. Similarly, the efficacy of the majority of standard motor interventions for promoting recovery after TBI is supported by rather limited evidence [6]. Therefore, investigation of other approaches to promote the recovery of motor impairments is essential. In this context, noninvasive brain stimulation (NIBS) appears to be an interesting option [26]. Transcranial Magnetic Stimulation (TMS) is delivered to the brain by passing a strong brief electrical current through an insulated wire coil placed on the skull. Current generates a transient magnetic field, which in turn, if the coil is held over the subjects head, induces a secondary current in the brain that is capable of depolarising neurons. Depending on the frequency, duration of the stimulation, the shape of the coil and the strength of the magnetic field, TMS can activate or suppress activity in cortical regions [27]. Another method of non-invasive brain stimulation is transcranial Direct Current Stimulation (tDCS) which delivers weak polarizing direct currents to the cortex via two electrodes placed on the scalp: an active electrode is placed on the site overlying the cortical target, and a reference electrode is usually placed over the contralateral supraorbital area or in a non-cephalic region. tDCS acts by inducing sustained changes in neural cell membrane potential: cathodal tDCS leads to brain hyperpolarization (inhibition), whereas anodal results in brain depolarization (excitation) [28,29]. Differences between tDCS and TMS include presumed mechanisms of action, with TMS acting as neuro-stimulator and tDCS as neuro-modulator. Moreover, TMS has better spatial and temporal resolution, TMS protocols are better established, but tDCS has the advantage to be easier to use in double-blind or sham-controlled studies [30] and easier to apply concurrently with behavioural tasks (for discussion of these methods, similarities and differences, see the review by Wagner et al. [31]). Despite their differences, both TMS and tDCS can induce long-term after-effects on cortical excitability that may translate into behavioural impacts that can last for months [32-35]. These long-term after-effects are believed to engage mechanisms of neural plasticity, rendering these techniques ideally suited to promote motor recovery particularly when combined with suitable behavioural interventions (for review, see [26,36,37]).

To date, two approaches have been tested. They are based on a model of interhemispheric rivalry between motor areas in the damaged and undamaged (intact) hemispheres. In essence, the model proposes that motor deficits are due to reduced output from the damaged hemisphere and excess inhibition of the damaged hemisphere from the intact hemisphere [26,38]. Thus, improvement may be possible by either up-regulating excitability of the lesioned motor cortex or down-regulating excitability in the intact motor cortex [26]. Enhancement of excitability can be achieved with either high frequency rTMS and anodal-tDCS. Suppression of excitability can be accomplished with either low-frequency rTMS and cathodal-tDCS. A growing body of evidence from small clinical trials has demonstrated the efficacy of both approaches to induce considerable changes on cortical excitability, which often correlate with relevant clinical gains in motor functions. However, most studies to date have examined the effects of NIBS without coupling it with any specific behavioural, physical or occupational therapy, and the functional benefits are often limited, inducing about 10–20% functional improvement in some single-session and longer-term therapeutic trials [37]. This is probably a suboptimal approach, as NIBS activates neural circuits in a non-specific way. Therefore given that NIBS and motor training are thought to share synergistic impacts on synaptic and network plasticity an emerging field of research is focusing on the possibility of coupling both therapies in order to achieve additive practical impact. The underlying principle of this approach is that practice of a motor task may be more effective at using the (surviving) neural mechanisms sub-serving training-dependent plastic changes if pertinent areas of the cortex are facilitated [38]. In addition, motor training can guide the activation of specific neural networks associated with the desired behavior. Considering for instance that many of the spontaneous plastic changes induced by a stroke, including phenomena of hyperexcitability, diminish after a few months [39-41], the therapeutic window of potential plastic changes for motor recovery seems to be limited. NIBS might be helpful to prolong this therapeutic window thus offering a greater opportunity for suitable physical and occupational therapies to promote functional recovery. Although preliminary, there is some recent encouraging evidence supporting the clinical validity of this approach.

### Mechanisms of NIBS to induce neuroplasticity

After a stroke affecting the motor cortex, cortical excitability is generally decreased in the affected primary motor cortex relative to the unaffected motor cortex. This might result from a shift in interhemispheric interactions, with increased transcallosal inhibition from the intact to the damaged motor cortex [41,42]. In this scenario, TMS and tDCS applied over the intact hemisphere allow safe cortical stimulation in humans in order to promote restoration of activity across bihemispheric neural networks and guidance towards more-adaptive plasticity [26,43].

TMS uses a rapidly changing magnetic field to induce electric currents via electromagnetic induction. A very brief high-intensity electric current is passed through a wire coil held over the scalp, this generates a magnetic field pulse which passes relatively unimpeded through the layers of tissue and bone and reaches the brain where secondary currents are induced. These secondary currents are induced in a plane parallel to the plane of the stimulation coil, which typically is held tangentially to the scalp, over the subject's head. Current direction and electric field distribution depend on output pulse shape of the stimulator and coil geometry respectively. The secondary current can be sufficient to depolarize cortical neurons, directly at their axon hillock or indirectly via depolarization of interneurons. Exactly which neural elements are activated by TMS and the mechanisms of neuronal stimulation remains unclear and might be variable across different brain areas and different subjects [27]. We know that when TMS is delivered over the primary motor cortex with adequate intensity, it induces efferent volleys along the corticospinal pathway [44]. Crucially, the therapeutic relevance of this technique is due to the long-term effects that occur after repeated stimulation. TMS delivered in a repetitive mode (rTMS) can indeed modulate cortical excitability beyond the duration of the rTMS trains themselves [45]. Depending on rTMS parameters, long lasting suppression or facilitation of cortical excitability can be induced: low-frequency rTMS (≤ 1 Hz) usually results in decreased cortical excitability [46], whereas at higher frequencies (>1 Hz) cortical excitability is usually increased [45]. It should however be noted, that this is an average effect across individuals, and yet there is substantial interindividual variability as well as intra-individual variability depending on the timing and exact location of stimulation [47,48].

In promoting stroke recovery, both, high frequency rTMS and low frequency rTMS have been tested and appear promising. For instance, Takeuchi et al. [49] and Fregni et al. [32] applied low-frequency rTMS to suppress activity in the contralesional (undamaged) hemisphere in chronic stroke patients: this suppressive protocol proved to be effective in reducing the transcallosal inhibition from the intact to the affected motor cortex [49] and increasing excitability of the lesioned motor cortex [32]. On the other hand, up-regulating the excitability of the lesioned M1 can also be successful. Talelli et al. (2007) reported that a single session of excitatory intermitted theta burst stimulation (TBS), consisting in delivering 3 pulses at 50 Hz, repeated at a rate of 5 Hz, increased MEP amplitude on the stroke side, with additional transiently improvement of motor behaviour [50]. By contrast, in the same study, continuous TBS of the unaffected motor cortex, which like low frequency rTMS suppresses excitability, did not change motor behaviour or the electrophysiology of the paretic hands [50]. Di Lazzaro et al (2008) obtained slightly different results. They showed that in acute stroke patients both intermittent TBS over the stroke hemisphere and continuous TBS over the intact hemisphere enhanced the excitability of the lesioned motor cortex and resulted in a functional benefit [51].

Despite these promising results, some limitations of TMS need to be noted. Critically, after stroke, there is a change in the local anatomy and the lesion evolves in time to formation of scar tissue and, particularly in the case of cortical damage, larger cerebrospinal fluid spaces. Because the conductance of cerebrospinal fluid (CSF) is 4 to 10 times higher than that of brain tissue, scar formation and larger CSF spaces modify the geometry and magnitude of the electric field induced by rTMS, and stimulation of the lesioned hemisphere can become difficult to predict unless careful modelling is done [52].

The mechanisms underlying long-term effects of TMS are incompletely understood, but they could be analogous to long-term potentiation (LTP) or depression (LTD) seen in the hippocampus after repeated activation of synaptic pathways [53-55]). In addition, modulation of neurotransmitter levels seems to be a contributing factor. The neurotransmitter systems involved include the inhibitory GABAergic system [56-58] as well as the excitatory glutamatergic system with activation of NMDA receptors [57]. TMS may result in changes in endogenous neurotransmitters (GABA and glutamate) and neuromodulators (DA, NE, 5-HT, ACh) which play a pivotal role in the regulation of the neuronal activity in the cerebral cortex (for review, [59]). A focal increase of dopamine in the striatum was indeed demonstrated in healthy human after sub-threshold 10 Hz rTMS applied to the ipsilateral primary motor cortex [60] or dorsolateral prefrontal cortex [61].

Another candidate mechanism by which rTMS may exert persistent effects is through gene induction. Actually, rTMS can modulate the expression of immediate early genes [62-64]. A single rTMS train increased c-fos mRNA in the paraventricular nucleus of the thalamus and, although to a lesser extent, in the frontal and cingulate cortices [64]. A longer treatment protocol (up to 14 daily sessions) could even induce an increase in c-fos mRNA in the parietal cortex of rodents [63] and an enhancement of BDNF mRNA in the hippocampus, the parietal and piriform cortices [65]. As suggested, BDNF is a neurotrophic factor that is critically linked to the neuroplastic changes [66] and might serve to index neuroplastic effects induced by rTMS [67].

The other main method of NIBS, tDCS, is a form of brain polarization that uses prolonged low-intensity electric current (1–2 mA) delivered to two large electrodes (usually 5 × 7 cm or 5 × 5 cm) to the scalp. To stimulate the primary motor cortex, usually one electrode is placed on the scalp over M1 and the other on the contralateral supraorbital area [68]. Alternatively, the reference electrode can be placed on the shoulder or another extra-cranial location. Reminiscent of the effects of repetitive TMS, tDCS can up- or down-regulate neural activity in the stimulated regions. Increased excitability of the underlying neurons occurs with anodal stimulation, while decreased excitability is seen after cathodal stimulation. With only 13 minutes of tDCS stimulation, effects on neural excitability outlasts the period of stimulation by up to 90 minutes [69]. In fact, the after-effects of tDCS appear greater than those induced by synchronous rTMS [68,70]. However, TBS or other, more sophisticated, asynchronous rTMS trains may significantly enhance and prolong the modulatory effects.

Again reminiscent of the effects of rTMS, tDCS-induced changes in cortical excitability are associated with changes in the excitability of inhibitory and facilitatory intracortical circuits: whereas anodal tDCS results in decreased intracortical inhibition and increased intracortical facilitation, cathodal stimulation induces opposite effects. In patients with chronic strokes, either anodal tDCS delivered to the lesioned M1 or cathodal tDCS delivered to the contralesional hemisphere can result in an improvement in motor functions [71-73].

tDCS does not stimulate axons and cause them to discharge action potentials, as TMS does. Rather, it most likely targets neuronal signalling by manipulating ion channels or by shifting electrical gradients which influence the electrical balance of ions inside and outside of the neural membrane; thus modulating the resting membrane threshold. Apart from membrane potential changes, chemical neurotransmission, either pre- or postsynaptically, may play a role in tDCS effects [74]. Some studies have aimed to clarify the cellular mechanisms of tDCS over the motor cortex [29,74]. For instance, the effects of the sodium channel blocker carbamazepine, the calcium channel blocker flunarizine and the NMDA-receptor antagonist dextromethorphane on tDCS-elicited motor cortex excitability changes were tested in healthy human subjects. Carbamazepine selectively eliminated the excitability enhancement induced by anodal stimulation during and after tDCS. Flunarizine resulted in similar changes. Antagonizing NMDA receptors did not alter current-generated excitability changes during stimulation, but prevented the formation of after-effects independent of their direction. Therefore, authors concluded that cortical excitability shifts induced during tDCS in humans appear to depend on membrane polarization, thus, modulating the conductance of sodium and calcium channels. In addition, the after-effects seem to be NMDA-receptor dependent. Recently, it was demonstrated that d-cycloserine, a partial NMDA-agonist, selectively potentiates the duration of motor cortical excitability enhancements induced by anodal tDCS [75]. Additionally, it was also suggested that the after-effects of cathodal tDCS include nonsynaptic mechanisms based on changes in neuronal membrane function [76]. Long term effects induced by tDCS may include built-up of new synapses, with mechanism of LTP and LTD critically involved. The glutamatergic system, in particular NMDA receptors [77], seems to be necessary for induction and maintenance of neuroplastic after-effect excitability enhancement and reduction induced by tDCS [74].

### Mechanisms of action of motor training in inducing plastic changes

After brain damage, there is substantial recovery with clearly delineated dynamics, resulting in a faster recovery in the acute and subacute stages, gradually levelling off as time progresses. In addition activity-dependent long-term modification of synaptic efficacy is associated with information storage in neural networks [78]. In fact, Neural plasticity changes evolve from the Hebbian synapse rule that states that individual synaptic junctions respond to activity (use) and inactivity (disuse) [79].

Motor training can promote plastic changes in injured motor networks even in a chronic stage of illness. However, simple interventions such as repetitive movement practice fail to induce profound plastic changes [80]. It appears that skill learning must be present to promote cortical plasticity [81]. In fact, most of the recovery of function after a stroke may represent actual relearning of the skills with the injured brain. Recovery mediated by training, like learning in healthy subjects, is usually task-specific and it differs from processes involved in compensation: whereas recovery of motor functions requires the recruitment of brain areas to generate commands to the same muscles as were used before the injury, compensation is instead based on the use of alternative muscles to accomplish the task goal [82]. Motor learning will lead first to strengthening of existing neural pathways, and second, to new functional or structural changes and thus expression of neuroplasticity [8].

The main mechanism underlying this relearning process after stroke involves shifts of distributed contributions across a specific neural network. Investigations in adult animals have revealed that motor learning can promote a plastic reorganization of motor maps in M1 with the representations of specific movements used to perform the motor task selectively expanding in the motor cortex at the expense of other areas not used for forelimb representations [10]. Similar results have been obtained in humans. For instance, the acquisition of new fine motor program induces an enlargement of the cortical motor areas targeting the muscles involved in the task, with an additional decrement of the activation threshold, as measured by means of TMS. Such map expansions parallel improvements in motor performance [83]. These results indicate that the cortex has the potential for rapid and large-scale functional changes in response to motor skill learning. One important issue is that an enlargement of a given neural network occurs at the cost of modifying another network and therefore with the theoretical risk of decreasing performance in another task. To date, this theoretical concern does not seem to cause any significant impairments in stroke subjects receiving intensive motor training.

Evidence for a long-term alteration in brain function associated with a therapy-induced motor recovery in neurological populations has also been provided. For instance, constraint-induced movement therapy (CIT) can significantly change cortical excitability measured by TMS in both affected and unaffected hemispheres. More specifically, CIT can result in an enlargement of the motor output map in the affected hemisphere, which is associated with a greatly improved motor performance of the paretic limb. A shift on the center of gravity of the output map in the affected hemisphere was also observed, indicating the recruitment of adjacent brain areas. Follow-up examinations up to 6 months after treatment showed that motor performance remained at a high level, whereas the cortical area sizes in the two hemispheres became almost identical, representing a return of the balance of excitability between the two hemispheres toward a normal condition [84]. These results are in line with PET and fMRI studies in recovered stroke patients showing that plastic changes taking place within the ipsilateral, noninfarcted hemisphere might contribute to the restitution of motor function [7,85-87]. A recent meta-analysis further underlines the positive impact of motor rehabilitation for the upper extremities, showing that practice-dependent recruitment of the ipsilesional hemisphere induces clear functional motor gains [88]. Increased engagement of the damaged hemisphere is expressed by either an increase in the area of the brain subserving the paretic arm movement, as shown by brain imaging techniques, and by greater signal strengths of physiological-functional measures (MEPs) within the sensorimotor cortex of the lesioned hemisphere [88].

Although motor training can lead to neurofunctional adaptation within a matter of minutes [89], long-term representational changes may take days [83] or weeks of practice [90]. Rapid changes are bound to be reflected in a less specific remodelling of network activity [91]. Instead, enduring change is reflected in, for example, augmented dendritic branching [92] and synaptogenesis [93], possibly provoked by specific gene induction [94,95]. Ultimately these processes result in an increase in the efficacy of synaptic transmission [96]. In strict analogy with the NIBS-induced after-effects, NMDA receptor activation and GABAergic inhibition are likely mechanisms operating in use-dependent plasticity in the intact human motor cortex and point to similarities in the mechanisms underlying this form of plasticity and long-term potentiation (LTP) [97]. LTP is associated with the proliferation of dendritic spines [98]. This morphologic change has been even found in homologous cortex opposite from the site of an experimental sensorimotor cortical lesion when the unaffected limb works to compensate for the paretic one [99]. This evidence suggests that the synaptic strength of horizontal connections in the motor cortex are modifiable and may provide a substrate for altering the topography of cortical motor maps during physical intervention based on motor learning.

### Combination of NIBS with motor training to enhance neuroplasticity and behavioural changes

As we have seen, motor learning and NIBS may share similar mechanisms of action for inducing neuroplastic changes in the human cortex. One possible conjecture then, is that their combination might mutually maximise their individual effects. Since learning processes are accompanied by cortical excitability shifts and by changes of synaptic efficacy and considering that the after-effect of NIBS is NMDA-receptor dependent, there is a possibility that cortical excitability changes induced by cortical stimulation could interact with ongoing motor learning process, improving learning-related NMDA-receptor strengthening. It is noteworthy that synaptic plasticity is bidirectional [100]. The basic idea is that the ongoing state of the cortex at the time of physical therapy can reinforce the long-term effects induced by motor practice. If so, the rationale of coupling NIBS and motor therapy is that it is possible to enhance or depress the response of a neural network to a form of stimulation, e.g. motor training, by previous priming it with a different form of stimulation, e.g. NIBS (and vice versa). Some experimental studies provide preliminary support to this hypothesis.

Animal studies using direct repetitive electric stimulation (ES) of the cortex – a technique that mimics rTMS and can alter cortical excitability as measured by cortical spreading depression (CSD) [101] have supported the importance of priming brain activity. CSD is an indicator of cortical excitability [102] characterised by alterations in cerebrocortical ion homeostasis in response to the direct stimulation of brain tissue. The alterations result in a wave of neuronal excitation propagating through the cortex followed by transient inhibition. It has been found that when active or sham 1 Hz ES was applied to Wistar rats preconditioned with active, sham or cathodal tDCS, a pattern suggestive of homeostatic mechanisms emerged [101]: 1 Hz ES that was applied alone or was preceded by cathodal tDCS, reduced CSD velocity whereas anodal tDCS followed by 1 Hz ES increased CSD velocity. Homeostatic effects have also been found in the effects of tDCS on paired associative stimulation (PAS) of human motor cortex [103] or by preconditioning of rTMS with tDCS [104]. However there is a fundamental difference when coupling two techniques of neuromodulation vs. coupling neuromodulation techniques with motor training; the latter might be better as it can focalize the effects to specific networks. In fact, several studies have explored the influence of coupling learning tasks with NIBS on motor and cognitive functions in healthy subjects. In one example, TMS synchronously applied to a motor cortex engaged in a motor learning task was shown to be effective in enhancing use-dependent plasticity. Healthy volunteers were studied in different sessions: training alone, training with synchronous application of TMS to the motor cortex contralateral or ipsilateral to the training hand, and training with asynchronous TMS. It was found that the longevity of use-dependent plasticity was significantly enhanced only by TMS applied in synchrony to the cortex contralateral to the training hand [105]. Carey et al. (2006) have obtained, however, different results: investigation of the effects of motor learning training, consisting in finger tracking with the right hand, unexpectedly showed that 1 Hz rTMS interfered transiently with motor performance when applied ipsilateral to the training hand but it had no effect when applied contralaterally [106]. In another tDCS study [107], the excitability of MT+/V5 and M1 was increased or decreased by anodal or cathodal tDCS while subjects were learning a visually guided manual tracking task. Accuracy of tracking movements was increased significantly by anodal stimulation, whereas cathodal stimulation had no significant effect on visual learning. Interestingly, the positive effect of anodal tDCS was restricted to the learning phase, suggesting a highly specific effect of the stimulation. Similar results were demonstrated for implicit motor learning in healthy human subjects [108], and in addition a recent study showed the beneficial effects of anodal tDCS over the posterior part of the left peri-sylvian area on language learning [109]. One important conclusion is that the effects are dependent on the site of stimulation, task and parameters of stimulation, therefore making difficult to generalize conclusions on these studies and also opening the possibility to induce detrimental effects when coupling these two intervention methods; therefore, it is critical to study the combination of these techniques before using it in clinical practice.

Originally, encouraging results have been found in animal investigations. Seminal experiments in animals have shown that coupled forced use of the paretic hand with implanted electrical stimulation to the ipsilesional M1 lead to significant behavioural improvements with large-scale expansions of the hand representation into areas previously representing proximal forelimb movements [80,110]. In a similar way, a recent prospective, randomized, multicenter study showed that in chronic stroke intensive motor therapy combined with invasive epidural electrode is associated with a significant improvement in motor function [111].

Although investigation with NIBS is still at the beginning, there are some very promising preliminary results. Khedr et al. (2005) have explored the effects of rTMS in patients with acute ischemic stroke as an add-on intervention to standard physical and drug therapies. rTMS was applied over the M1 of the stroke hemisphere for 10 days. rTMS consisted in ten 10-second trains of 3-Hz stimulation with 50 seconds between each train [112]. The motor treatment consisted in the passive limb manipulation, increasing by the end of the first week to more active movements if patients improved function. Treatment effects were measured with clinical scales and neurophysiological measurements – i.e., resting motor threshold (RMT) of healthy side, and motor evoked potentials (MEPs) of healthy and hemiplegic sides. On every scale, patients' motor scores in the active rTMS group had a significant greater improvement as compared with sham rTMS, leading to a higher percentage of independent patients and a higher percentage of patients having only mild disability by the time of the follow-up assessment, after 10 days from the end of the treatment. However, no effect was seen in patients with massive middle cerebral artery infarcts. 14 out of 21 patients in the real rTMS group recovered MEPs; although MEPs tended to improve more in the real rTMS group, this was not significantly different from the sham group. In addition, no correlation between clinical recovery and changes in MEP was found [112].

In other clinical trial [113], patients with chronic hemiparetic stroke practiced a complex, sequential finger motor task using their paretic fingers either after receiving high-frequency (10 Hz, repeated 8 times) or sham rTMS over the primary motor cortex (M1) of the damaged hemisphere. Changes in the behavior and corticomotor excitability before and after the intervention were examined by measuring the movement accuracy, the movement time, and the MEP amplitude. The authors found that rTMS induced a significantly larger increase in the MEP amplitude than sham rTMS; this corticomotor excitability change was associated with enhanced motor skill acquisition.

Rather than trying to enhance the cortical excitability of the damaged motor cortex, Takeuchi et al (2008) explored the effect of inhibiting the contralesional motor cortex in chronic patients [114]. Patients were randomly assigned to receive either a sub-threshold rTMS over the unaffected hemisphere (1 Hz, 25 minutes) or sham stimulation and all patients performed a pinching task after stimulation. Compared with sham stimulation, rTMS induced an increase in the excitability of the injured motor cortex and an improvement in acceleration of the affected hand. The effect of motor training on pinch force was also enhanced by rTMS. Such improvement was stable at the follow-up examination, one week after the intervention [114]. Another study [115] assessed the efficacy of low-frequency 1 Hz rTMS combined with voluntary muscle contraction (VMC) on corticospinal transmission, muscle function, and purposeful movement early after stroke. rTMS consisted of 5 blocks of 200 1-Hz stimuli (using an interblock interval of 3 minutes), applied to the lesioned hemisphere. The treatment was given for 8 working days. The motor training task in this study was VMC – the paretic elbow was repeatedly flexed/extended for 5 minutes. The main finding was that in patients who underwent the rTMS combined with VMC, motor-evoked potential frequency increased 14% for biceps and 20% for triceps; whereas, with Placebo rTMS plus Placebo VMC, motor-evoked potential frequency decreased 12% for biceps and 6% for triceps.

Negative findings have also been reported. A recent study indeed did not prove the usefulness of combing rTMS stimulation with a standard motor therapy [116]. Here, chronic stroke patients undergoing ten days of constraint-induced therapy (CIT) for upper-limb hemiparesis, which was combined with 20 Hz rTMS (stimulus train duration of 2 secs, intertrain interval of 28 secs.) or with sham rTMS of the affected M1. Primary outcome measures to assess change in upper-extremity function were the Wolf Motor Function Test (WMFT) [117] and the Motor Activity Log (MAL)-Amount [118]. Secondary outcome measures included the MAL-How Well and the Box and Block Test (BBT) [119] and MEP threshold. The results showed that, regardless of the rTMS intervention, participants demonstrated significant gains on the primary outcome measures and on secondary outcome measures, further supporting the efficacy of CIT. Indeed, although a significant decrease in motor threshold for subjects receiving rTMS was found, which was not observed after sham rTMS, this increase in the excitability of the motor system did not translate into a clinically evident outcome. Ceiling effects and outcome measures might have contributed to these findings.

tDCS is another technique associated with a significant beneficial effect on motor recovery after stroke. Although its beneficial effects on motor function have been shown by several small studies [26,32,43,71,72,120], actually there are very few clinical trails of the potential adjuvant of this technique to physical therapy. Yet, tDCS might be a more suitable tool to enhance the effects of motor training as it offers several advantages as compared with TMS in a rehabilitation setting. For instance, whereas rTMS has to be delivered in a off-line paradigm and it usually precedes the behavioural intervention, the portable use of tDCS allow to deliver the cortical stimulation during the motor training. Moreover, tDCS modulatory effects last longer as compared to rTMS – for example, 13 minutes of stimulation changes brain excitability for up to 90 minutes [69]. Finally, due to its physiological effect on the membrane resting potential, tDCS could to be more appropriate for priming motor neural network for subsequent stimulation with tDCS. Hummel et al (2005) have recently explored the effects of tDCS on skilled motor functions in chronic stroke patients [121]. Anodal tDCS was delivered for 20 min to the affected hemisphere during the execution of the Jebsen-Taylor Hand Function Test (JJT), a widely used assessment of functional hand motor skills. Active anodal tDCS was associated with improvements in motor function of the paretic hand. The magnitude of tDCS-induced improvement in JTT was approximately 11.75% (+/- 3.61%) and persisted for more than 25 min after the stimulation ended. However, patients' performance returned to baseline levels after 10 days of the end of stimulation [72]. In another preliminary report in chronic stroke, anodal tDCS (1.5 mA) was combined with robot-assisted arm training (AT) [122]. Over six weeks, patients received 30 sessions of 7 min tDCS integrated into 20 min of AT. Arm function of three out of ten patients (two of them with a subcortical lesion) improved significantly, as measured by the Fugl-Meyer motor score. In the remaining seven patients, all with cortical lesions, arm function changed little. However, this study lacked an adequate control group and it included a small number of patients, who were still in the phase of spontaneous recovery; therefore no definite conclusions can be made.

Overall, the data discussed above provide some encouraging information supporting the proposal that NIBS might optimize the effect of standard physical therapy under certain circumstances. Beyond the obvious need for further clinical trials to corroborate the validity of this approach, attention must be directed in understanding the optimal way to combine motor training with NIBS. Crucially the next step is to determine the best parameters required to optimize the conditioning effects of NIBS on motor therapy, as well as the exact temporal window during which NIBS can be delivered in order to modulate brain plasticity and enhance the effects of the motor training.

### How brain stimulation should be used in combination with motor training – methods of optimizing functional improvements

Given the limited number of clinical trails that have assessed the efficacy of combining NIBS with physical therapy, any prediction of the clinical utility of this approach remains speculative. Although further investigations are needed to make any relevant clinical consideration, some reflections can be delineated in order to make an optimal use of this approach in the near future. So far, the best option in order to optimize the effects of coupling NIBS and motor therapy still needs to be explored but it likely may depend on different factors, as the stages of illness (e.g. acute versus chronic), the type of motor training, the site of stimulation, the timing of stimulation in relation to physical intervention, baseline cortical activity and the technique of NIBS used. An essential issue to take into account when applying these NIBS protocols to a damaged human brain is related to the concept of homeostasis – that is the human's brain ability to regulate changes in synaptic plasticity as to avoid drastic changes in its function. Therefore homeostasis is likely to respond definitively and forcefully to artificial and functionally non-specific changes in network activity such as those probably induced by NIBS [123]. Homeostatic plasticity (i.e., the dependency of the amount and direction of the obtainable plasticity from the baseline of a neuronal network) is increasingly recognized as regulatory mechanism for keeping neuronal modifications within a reasonable physiological range. Homeostasis provides a means for neurons and circuits to maintain stable functions in the face of perturbations such as activity-dependent changes in synapse number or strength [124]. In this regard, recent experimental works emphasize the importance of homeostatic plasticity as a means to prevent destabilization of neuronal networks that could operate in neurorehabilitative settings [124,125]. In particular, as advised by Thickbroom (2007), the influence of homeostatic mechanisms cannot be overlooked either during or after NIBS interventions: homeostatic mechanisms could be a crucial factor in repeat interventions, as are sometimes employed in NIBS protocols, or for intervention protocols of longer duration in which they may begin to act during the intervention itself. They could be one of the main factors that limit the magnitude and duration of post-TMS effects. For instance, NIBS could evoke compensatory regulatory mechanisms, which are a part of the process of maintaining normal brain function. On the other hand, activity-dependent forms of plasticity, even those incorporating LTP and LTD mechanisms, are inherently unstable due to positive feedback [123]. Thus, the successful implementation of NIBS as adjuvant strategy to physical therapy should rely on an improved understanding of the underlying plastic mechanisms and their functional interaction with activity-induced plasticity. For instance, a challenging issue is the time of the NIBS intervention relative to the motor task. As seen above, when combing to a motor training, so far NIBS has been usually delivered just before the task. However, functional therapies could in principle be implemented at different phases in conjunction with a NIBS intervention. NIBS preceding the motor training could potentially prime functional networks for the physical intervention. Instead, NIBS simultaneously applied during a behavioural intervention might preferentially interact with the networks selectively recruited by the ongoing task. Even the application of NIBS after motor training could be a potential choice; the underlying rationale of this approach is that, after the modulation induced by the motor therapy, a further modulation of cortical excitability might selectively build up the activity-dependent activation of a given network and promote its functional stabilization. It is not completely unlikely that even after excitability has returned to baseline, perhaps due to homeostatic regulation, NIBS could still be functionally beneficial [123]. Here, NIBS could be influential for driving longer-term consolidation of new network patterns. The choice of the more suitable time window for NIBS intervention likely needs careful examination in order to exclude maladaptive cortical responses, which could interfere with or even suppress the effects of the behavioural therapy. For instance, an excitability modulation induced by tDCS during the performance of a motor task might be best suited to improve motor learning than tDCS administered prior of learning or motor behaviour [126,127]. This is because, during tDCS not only NMDA receptors, but also calcium channels are modulated, while the after-effects of tDCS are achieved by modifications of NMDA receptors alone [29]. Since intracellular calcium concentration is important for LTP induction [128] enhanced transmembrane calcium conduction, as probably achieved during anodal tDCS, might improve learning processes. On the other hand, a pure modulation of synaptic strength prior to learning might compromise performance, due to homeostatic or defocusing effects. Therefore, administering tDCS during, and not before, motor learning might be the best strategy to improve the effects of physical therapy [126].

The parameters of stimulation – such as number of stimulation sessions, frequency, intensity and site of stimulation – need to be taken in consideration. Relative to the duration of the cortical stimulation, it is worth mentioning that NIBS interventions have relatively short-lived after-effects compared to experimental LTP/LTD or to the duration needed for any clinically relevant functional improvement. However, repeated sessions of NIBS may have cumulative effects; perhaps due to these cumulative effects, several sessions of NIBS are usually associated with greater magnitude and duration of behavioural effects [129]. This has been also reported in clinical trials in stroke patients, in which stimulation with rTMS for 10 days can indeed induce a long-lasting improvement of motor behaviour that lasted for 10 days after the end of stimulation [32,112]; similarly, cathodal tDCS applied over 5 consecutive days is associated with a cumulative motor function improvement that lasts up to 2 weeks after the end of stimulation. However, interesting, this effect is not observed when sessions are applied weekly instead of daily [73]. In fact, multiple stimulation sessions are required in order to induce a significant manipulation in synaptic efficacy [130]. Thus, future clinical trails need to take into account that only prolonged and consecutive sessions of NIBS can translate into a long-lasting functional gains in stroke patients.

Until now physical therapy has been largely combined with NIBS applied to the motor cortex; nonetheless, other brain areas might be involved in motor recovery. For instance, higher levels of contralesional activity in prefrontal and parietal cortices appear to be predictive of a slower motor recovery, suggesting a possible negative role of activity in these areas of the intact hemisphere in functional restoration [38,131]. If so, suppression of such activity with NIBS might be a valuable intervention. Thus, modulation of excitability in areas beyond the primary motor cortex should be also taken into account as potentially interacting with the damaged motor areas, driving their activity-dependent activation. In patients with TBI, given their additional attentional impairments which negatively impact the efficacy of standard motor therapies [132], a modulation of attentional networks might enhance the responsiveness to standard motor rehabilitation.

Another controversial issue is related to the side of stimulation. It is still unclear whether it is better to suppress activity in the undamaged hemisphere or increase activity in the perilesional cortex. To date only one study, using tDCS, has directly compared the effectiveness of down-regulating the contralesional hemisphere with facilitation of the stroke hemisphere in patients with motor stroke; both approaches were found to be equally effective, with slightly greater improvement after suppression of the intact hemisphere [71]. However this was a small study and patient selection might have played a significant role. It is not yet known whether this is also true for rTMS. At least it appears that application of excitatory rTMS protocols to the stroke hemisphere is safe and does not increase the risk of provoking a seizure [133]. In any case, it is likely that rather than a global modulation of one or another hemisphere, more targeted, focal modulation of activity in selected cortical regions of each hemisphere might be desirable. Furthermore, the application of different strategies in different phases following the brain insult might be needed. Finally, it is worth remembering that currents induced by NIBS in the lesioned brain can be perturbed by anatomical changes which can render the neuromodualtory effects less predictable [52].

Importantly, the effects of NIBS are also task dependent; therefore it is possible that some motor tasks are more susceptible to modulation by NIBS than others. If so, the choice of the motor training task might be a critical determinant for the success of the therapy.

Overall, if guided by a careful consideration of the underlying mechanisms, the combination of NIBS with functional therapies has the potential to drive plastic changes in brain-damaged patients. This might in turn promote remarkable clinical gains in motor functions that otherwise could not be achieved by administering NIBS or motor treatment alone. Clearly, further investigation is warranted to address the overall utility of NIBS as an adjuvant to stroke rehabilitation, and the optimal strategy to combine the two interventions in order to maximize their functional interaction.

## Conclusion

The uninjured tissue may be particularly receptive to modulation by various external tools including behavioral training and neuromodulatory approaches such as noninvasive brain stimulation. Given that both strategies, motor learning and cortical stimulation, have some similarities in their mechanisms of action, such as both induce similar changes in the local excitability in the lesioned and contralesional motor cortical area associated with long-lasting after-effects, their combination might be more beneficial than their use alone. In fact, brain stimulation can prime cortical excitability for a subsequent motor training task therefore optimizing processes of motor learning involved in standard rehabilitation therapies, leading to more pronounced and longer lasting functional gains. Some preliminary evidence seems to support this view. However, other studies failed to demonstrate a significant effect of brain stimulation as an adjuvant to standard motor therapy. In the future, the successful implementation of combined NIBS and motor therapy will critically rely on improved understanding of their functional interactions and associated effects on neural plasticity. Greater understanding of the mechanisms of action of each approach is necessary in order to optimize their combined use in rehabilitation and realize the promise of a more effective means to promote functional recovery after brain injury.

## Competing interests

The authors declare that they have no competing interests.

## Authors' contributions

NB, APL and FF conceived the initial idea. NB and FF wrote the first draft and all authors revised and approved the final manuscript.
